# Interrogating structural inequalities in COVID-19 mortality in England and Wales

**DOI:** 10.1136/jech-2021-216666

**Published:** 2021-07-20

**Authors:** Gareth J Griffith, George Davey Smith, David Manley, Laura D Howe, Gwilym Owen

**Affiliations:** 1 MRC Integrative Epidemiology Unit, University of Bristol, Bristol, UK; 2 Population Health Sciences, Bristol Medical School, Bristol, UK; 3 School of Geographical Sciences, University of Bristol, Bristol, UK; 4 Department of Public Health and Policy, University of Liverpool, Liverpool, UK

**Keywords:** social inequalities, COVID-19, mortality, epidemiological methods, health inequalities

## Abstract

**Background:**

Numerous observational studies have highlighted structural inequalities in COVID-19 mortality in the UK. Such studies often fail to consider the hierarchical, spatial nature of such inequalities in their analysis, leading to the potential for bias and an inability to reach conclusions about the most appropriate structural levels for policy intervention.

**Methods:**

We use publicly available population data on COVID-19-related mortality and all-cause mortality between March and July 2020 in England and Wales to investigate the spatial scale of such inequalities. We propose a multiscale approach to simultaneously consider three spatial scales at which processes driving inequality may act and apportion inequality between these.

**Results:**

Adjusting for population age structure and number of local care homes we find highest regional inequality in March and June/July. We find finer grained within region inequality increased steadily from March until July. The importance of spatial context increases over the study period. No analogous pattern is visible for non-COVID-19 mortality. Higher relative deprivation is associated with increased COVID-19 mortality at all stages of the pandemic but does not explain structural inequalities.

**Conclusions:**

Results support initial stochastic viral introduction in the South, with initially high inequality decreasing before the establishment of regional trends by June and July, prior to reported regionality of the ‘second-wave’. We outline how this framework can help identify structural factors driving such processes, and offer suggestions for a long-term, locally targeted model of pandemic relief in tandem with regional support to buffer the social context of the area.

## Background

Inequality in COVID-19 outcomes has been a matter of keen interest in the UK since the early stages of the pandemic.^1 2^ The ‘second wave’ of SARS-CoV-2 infection and subsequent COVID-19-related illness across the UK has been characterised by a strong regionality and an emergent North-South divide in mortality which both reflected and exacerbated existing social inequalities.^3 4^

An enormous literature has evolved over the course of the pandemic on individual predictors of COVID-19-related outcomes.^5 6^ A smaller body of work has also emerged looking at area-level predictors of COVID-19 outcomes and exposures (eg, ref 7–9). Such spatial investigations have, however, largely focused on COVID-19 case status which is subject to temporally varying testing priorities.^10^ Population testing was limited in the early pandemic, when testing was more likely for severely ill or hospitalised individuals. We therefore assume data on COVID-19-related mortality are less likely to be severely biased than case numbers as, conditional on life-threatening conditions, COVID-19 status will be more accurately reported among those in critical care.

We conceptualise COVID-19 mortality as a function of two processes: the risk of infection, and the risk of death given infection. Each of these is unlikely to have a uniform spatial distribution. Spatial analyses of aggregated mortality data are critical to epidemiological triangulation efforts as they give (fallible) measures of population parameters not subject to the same selection processes as individual-level analyses.^11^ We suggest predictors of aggregate mortality may primarily reflect risk of infection, as evidence does not suggest viral evolution has affected disease prognosis^12 13^ and restricted migration patterns (given suppression measures) are unlikely to explain short-term monthly differences within an age-adjusted population structure.

**Figure 1:**
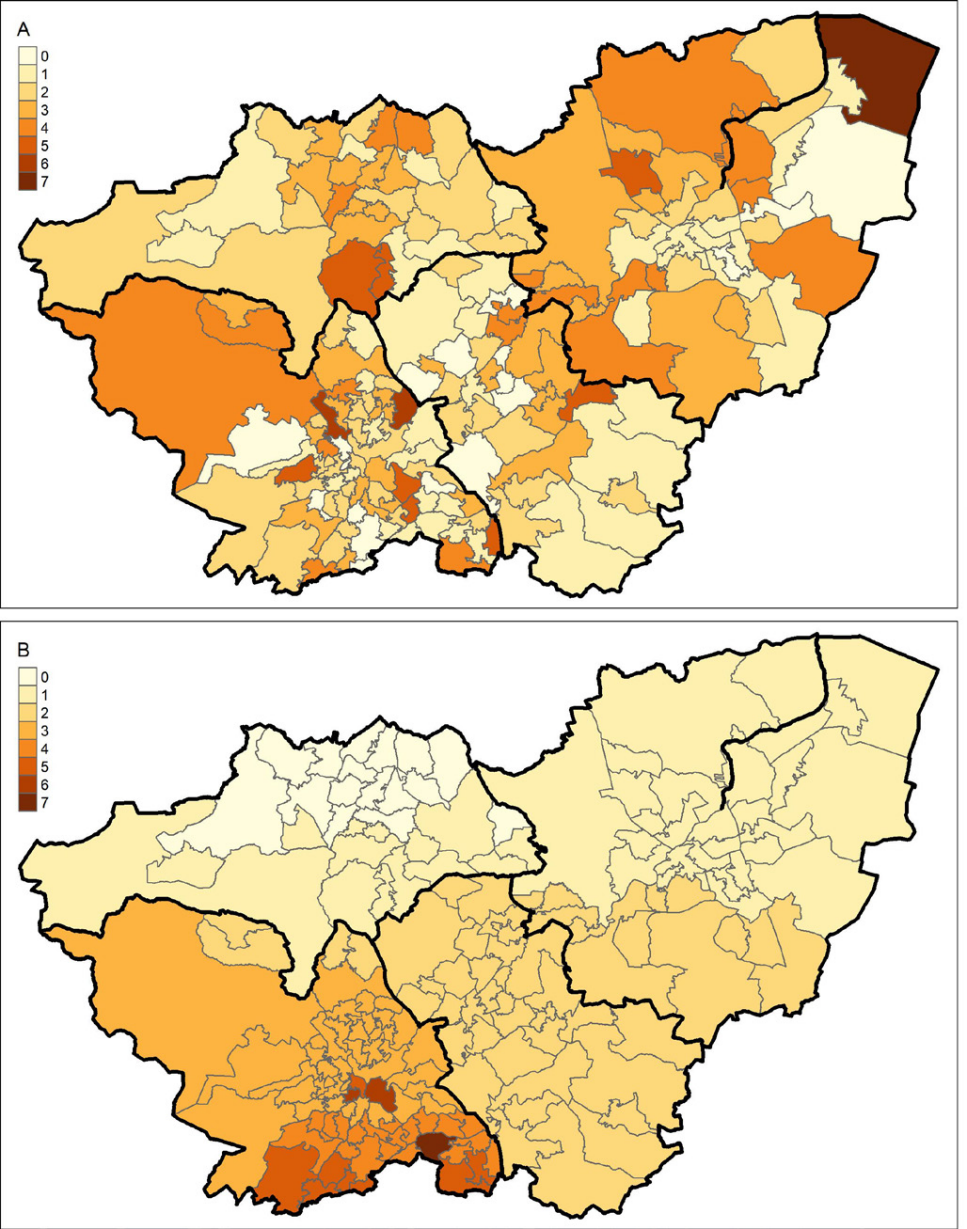
A map of South Yorkshire Middle-Layer Super Output Areas (MSOAs), with four larger local authority districts (LADs) highlighted by darkened bounds. (A) Shows a hypothetical scenario in which the large majority of inequality is truly between MSOA, within LAD. (B) Shows a hypothetical scenario in which the large majority of inequality is truly between LAD, with very little within-LAD variation.

Investigation into COVID-19 inequalities has largely been restricted to analyses at a single, aggregated spatial scale, predominantly governmental office region (GOR; n=10 in England and Wales) (eg, ref 14) or smaller Middle-Layer Super Output Areas (MSOAs; n=7201 in England and Wales) (eg, ref 8 15 16). However, limited spatial scope may bias results of such aggregated analyses as, when higher level clustering is omitted from a model, higher level phenomena are necessarily expressed at the included spatial scale.^17^ Restricting policy-relevant ecological analysis to a single geographical, administrative scale risks overstating the importance of the included scale and missing structural exposures at which to target interventions and resources. Locally targeted resource allocation presents a key UK policy intervention which may be acted on to reduce health inequalities (eg, ref 18). We provide a simple vignette outlining this problem in box 1 and figure 1. Thus, the higher order spatial nature of processes driving between area inequalities in COVID-19 mortality is currently underinvestigated. To better understand area-level mortality differences, we must interrogate regional COVID-19 mortality inequality net of lower level variation, and investigate at what stage of the pandemic it emerged.

Box 1 **: Why care about spatial clustering?**

The degree of spatial inequality which exists with respect to a given phenomenon depends on the spatial scale at which the question is asked.^42 43^ This is often referred to as the modifiable area unit problem (MAUP). A guided explanation of this is provided in figure 1.

The map displays South Yorkshire. The smaller areal units are Middle-Layer Super Output Areas (MSOAs), and the areas outlined in thicker black lines are local authority districts (LADs). In a single-level model of MSOA mortality, the two maps below are identical. More explicitly, Poisson overdispersion (and residual error variation in linear regression) is assumed to be spatially random. This assumption is commonly violated, as it is in figure 1B, where residuals are clearly spatially structured.

To meaningfully decompose this variation, we must consider both between and within interpretations of inequality. In figure 1A, the majority of variation is *within LAD*, *between MSOA*. Net of any higher level geography—this is close to spatially random, and we may assume that any spatial processes occurring in the data are at the MSOA level. In figure 1B, however, most variation is *between LAD*, with very low *between-MSOA, within-LAD* variation.

If we only take account of the MSOA clustering (for instance, by only including MSOA mortality counts) we would conclude, consistently across both plots, that MSOA is the level at which spatial processes are most likely to be occurring. Thus, in the scenario in figure 1B we would fail to recognise that LAD context may inform a structural exposure. However, if we simultaneously account for both MSOA and LAD, we can investigate the relative importance of each. Interrogating the relative importance of multiple geographical levels may better inform the likely exposures driving spatial inequalities, and hence inform potential interventions.

**Figure 6:**
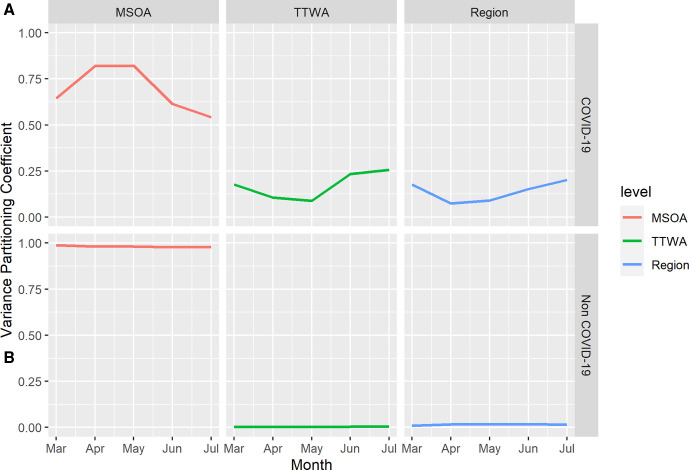
Unadjusted model monthly variance partitioning coefficients (VPCs) for three administrative scales for COVID-19-related mortality (A) and non-COVID-19 mortality (B). VPC units are proportions. MSOA, Middle-Layer Super Output Area; TTWA, Travel to Work Area.

### Overview

In this article, we use publicly available MSOA-level mortality for the period of March to June 2020 to unpack the geographical scale at which inequality in COVID-19 mortality was most keenly experienced. We rerun all analyses using non-COVID-19-related mortality figures to contextualise inference about whether observed spatial processes are unique to COVID-19. Having established area-level differences, we explore area-level characteristics which may explain these inequalities.

We structure our analysis via the following three research questions:

1. What is the geographical scale at which mortality inequality (both COVID-19 related and non-COVID-19 related) is most strongly expressed between March and July 2020?
2. Is spatial patterning consistent over the study period?
3. To what extent are these inequalities explained by local area deprivation?

## Methods

### Data

All data used for this analysis are publicly available from the Office for National Statistics (ONS), Environmental Systems Research Institute (ESRI) or derived data sets made available in existing published research.

### Outcome

Mortality data are counts of MSOA-level mortality from COVID-19 and non-COVID-19 sources.^19^ These data provide provisional counts of number of deaths involving COVID-19 (defined by the ONS and UK government as COVID-19 being mentioned on the death certificate^19 20^) for each MSOA in England and Wales between 1 March and 31 July 2020. They also provide a total count of deaths from which the number of non-COVID-19 deaths can be calculated.

### Structural levels

Investigating higher level spatial inequalities requires specification of plausible administrative, geographical scales at which mortality inequalities may be expressed. We select three analytical levels, chosen to represent plausible scales for spatial process, and also pragmatic levels for intervention.

The lowest level unit is the MSOA (n=7201), a commonly used parameterisation of local neighbourhood exposure relevant to COVID-19 transmission.^7 8^ We assume local neighbourhood scale captures neighbourhood migration and consumption patterns. MSOAs are hierarchically nested within ONS Travel to Work Areas (TTWAs, n=173). These are designed to approximate local labour market areas, and thus represent commuting patterns to local cities, a highly relevant context for COVID-19 transmission.^21 22^ Finally, each TTWA is nested within one of 10 GORs. These provide an intuitive representation of the macroscale health exposures which have been reported to predict COVID-19 mortality throughout the pandemic, particularly throughout summer 2020.^2 14^

Due to the relative infrequency of TTWA use in analyses we also rerun models using local authority districts as a sensitivity analysis for spatial parameterisation (see online supplemental figures 1 and 2). We also test the importance of a cross-classified unit using National Health Service Sustainable Transformation Partnerships (online supplemental figures 3 and 4).

10.1136/jech-2021-216666.supp1 Supplementary data

### Covariates

Older age has been established as the strongest individual predictor of COVID-19-specific mortality,^5 23 24^ with 74% of COVID-19 deaths in the period March to July being among those over 75.^19^ Care home population has also been suggested the strongest area-level predictor of COVID-19 mortality.^8 15^ We wish to estimate spatial inequalities between the average units at each geographical context, net of these known risk factors, so adjust for them in all analyses. ONS midyear population estimates for 2019 are used to account for MSOA age structure.^25^ MSOA-level care home data are taken from the open-access ESRI COVID-19 dashboard Geolytix data set which was made available for COVID-19 researchers. Care homes are included as raw counts at MSOA level.

We also wish to see whether local deprivation-related exposures explain any structural inequalities we observe. Local deprivation is associated with increased overcrowding, service industry jobs and intergenerational housing, which have been shown to be relevant exposures for COVID-19 mortality.^1 8^ To estimate contextual effects between similarly deprived units, we adjust for the Index of Multiple Deprivation (IMD) as an indicator of the intersecting disadvantage shown to be associated with worse COVID-19 outcomes.^5 7^ As the IMD is country specific we would not be able to compare England and Wales, so we use the equivalised UK IMD.^26^ This functions as an area-level indicator of disadvantage comparable across UK countries. Local healthcare was severely strained over the study period, so we decompose deprivation into between and within geographical components (ie, regional average IMD, TTWA minus region IMD, MSOA minus TTWA IMD). This offers us insight into whether between-area differences in IMD are similarly predictive of excess mortality as within-area differences. Between and within elements of IMD are standardised. As these data were generated from 2015 IMD results for UK countries we rerun analyses excluding Wales and using 2019 IMD scores (see online supplemental figures 5 and 6).

### Statistical analysis

To analyse multiscale inequality, we specify multilevel Poisson models with a geographically invariant, month-specific log offset. The offset term indicates expected COVID-19 mortality and is calculated by taking the total COVID-19 mortality across England and Wales in each month and dividing by the total population. This is then multiplied by MSOA population to give an expected count were mortality rates spatially invariant. The same was done for non-COVID-19-related mortality. Models were stratified by mortality classification and were run for both COVID-19 and non-COVID-19-related mortality. Covariates were interacted with a categorical month term to allow effects to vary over time (full detail on model specification in online supplemental material).

Any overdispersion in random coefficients over and above this spatially consistent offset can be used to infer clustering, or spatial inequality.^27^ We summarise the relative contribution of level-specific variance using variance partitioning coefficients (VPCs), which estimate the proportion of supraindividual, unexplained heterogeneity present at each structural level.^28 29^ We summarise the variation in random coefficients at a given level using median rate ratios (MRRs).^29 30^ Transforming the variance from a typical interpretation (exp(σ)) allows for comparisons between standardised rates, without needing to consider effects in terms of an SD increase in random effects:

$$MRR=e^{\sqrt{\left(2*\sigma^{2}\right)}*0.6745}$$

The MRR is interpreted as the median relative change in the mortality rate between randomly sampled pairs of lower level units within a higher level unit.^29 30^ For instance, an MSOA MRR of 1 would imply that there is no difference between risks of death in different MSOAs within the same TTWA. An MSOA MRR of 2 implies that in general, comparing between MSOAs where all else is equal (ie, within the same higher level unit, and balanced covariates), we would expect one of the MSOAs to have a mortality rate double that of the other. Where multiple higher levels are included in a model, it is important to note that MRRs are an estimated net of one another, such that inequality at each level is estimated separately from that in another.

We use Markov chain Monte-Carlo (MCMC) estimation for all models. Restricted iterative generalised least squares estimates were taken as starting values. After a discarded burn-in of 50 000 iterations, all models were run for 500 000 iterations. Credible intervals for all estimated quantities are the 2.5th and 97.5th percentiles of posterior parameter distributions. All models were run in MLwiN V.3.09.

## Results

**Figure 2:**
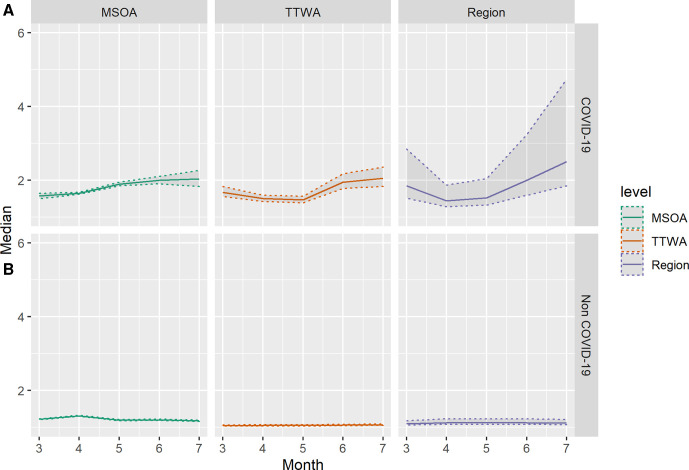
Estimates of median COVID-19 (A) and non-COVID-19 (B) median mortality rate ratio across three administrative scales, by month from March to July 2020. Shaded areas indicate 2.5th and 97.5th percentile credible intervals of posterior parameter distributions. MSOA, Middle-Layer Super Output Area; TTWA, Travel to Work Area.

Results are presented for COVID-19 and non-COVID-19 mortality. All models include fixed effects adjustment for MSOA age structure and number of care homes. Our first and second research questions regard the spatial and temporal variation in relative mortality risk over the study period and the relative contribution of each spatial scale. Monthly mortality counts are provided in online supplemental table 1. Results for the first pair of models are presented in figure 2.

Figure 2A illustrates the development over time of MRRs across different spatial scales. The COVID-19 mortality MRR in July for MSOA is 2.03 (95% CI 1.83 to 2.27), suggesting within-TTWA, between-MSOA differences typically imply a doubling of the rate of COVID-19 mortality. We see considerable temporal heterogeneity, with inequality at all scales increasing after April and March. This coincides with a decline in national mortality rates (see online supplemental table 1). Regional MRR is largest in July (MRR 2.51, 1.85–4.74), having declined in April as mortality rates increased, where largest inequalities were seen within TTWA, between MSOA.

The contrast with figure 2B is stark. Mortality rates for non-COVID-19 mortality are higher than those for COVID-19 mortality, yet they are far more spatially random, the highest rate ratio between areas at any of the scales is 1.32 (95% CI 1.30 to 1.33) at MSOA level in April. Inequality over and above that predicted by stochastic variation is only seen in April for non-COVID-19 mortality.

**Figure 3:**
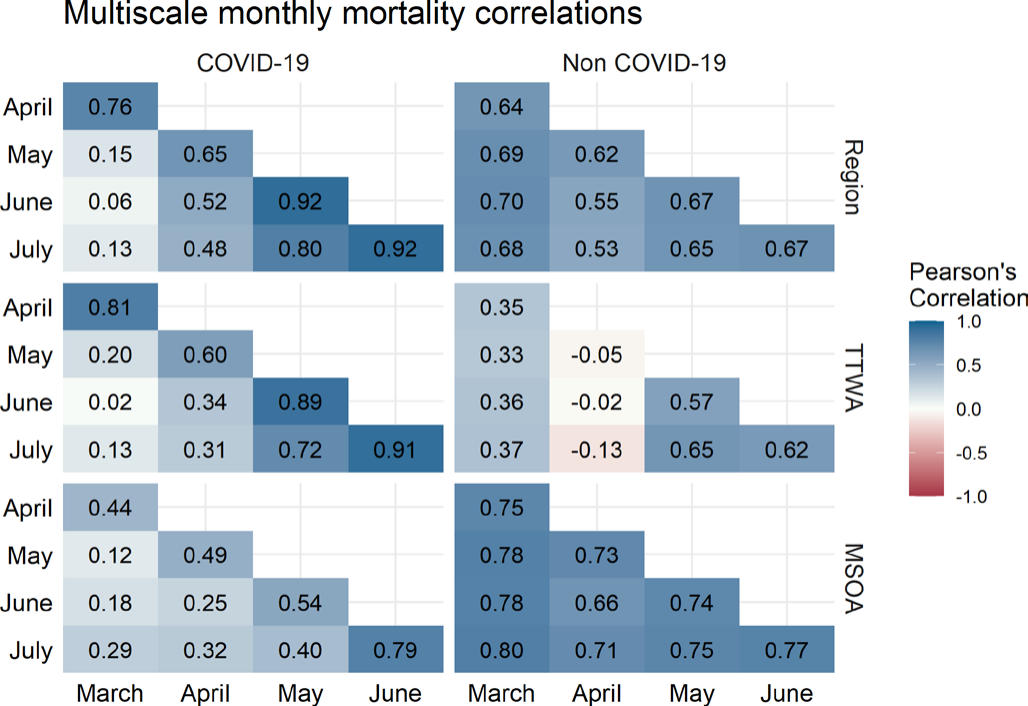
Correlation between month-specific structured residuals at three administrative scales for COVID-19 mortality (left) and non-COVID-19 mortality (right). Colour indicates magnitude and sign of correlation. MSOA, Middle-Layer Super Output Area; TTWA, Travel to Work Area.

Spatial inequalities in COVID-19 mortality have clearly not been temporally stable, but we are also interested in whether the residual patterning of mortality is consistent over time. That is, whether regions with high mortality in March have high mortality in subsequent months. figure 3 illustrates the correlation between area-level residuals from March to July.

Correlations are broadly larger between adjacent months. However, this is not temporally consistent and we see area-level trends start to emerge, for instance, the highest correlation is between June and July for all levels for COVID-19 mortality. Excluding TTWA, correlations for non-COVID-19 deaths are uniformly high, suggesting that the small amount of clustering we see in non-COVID-19 mortality is highly temporally autocorrelated. TTWA-level non-COVID-19 mortality residuals are low, and suggest unusual spatial patterning in April. MSOA COVID-19 mortality correlations increase consistently over the study period.

**Figure 4:**
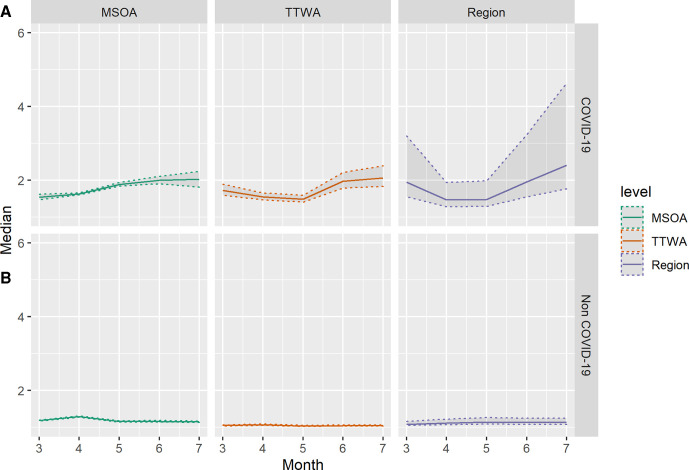
Estimates of median COVID-19 (A) and non-COVID-19 (B) median mortality rate ratio across spatial scales after inclusion of local area deprivation, by month from March to July 2020. Shaded areas indicate 2.5th and 97.5th percentile credible intervals of posterior parameter. MSOA, Middle-Layer Super Output Area; TTWA, Travel to Work Area.

**Figure 5:**
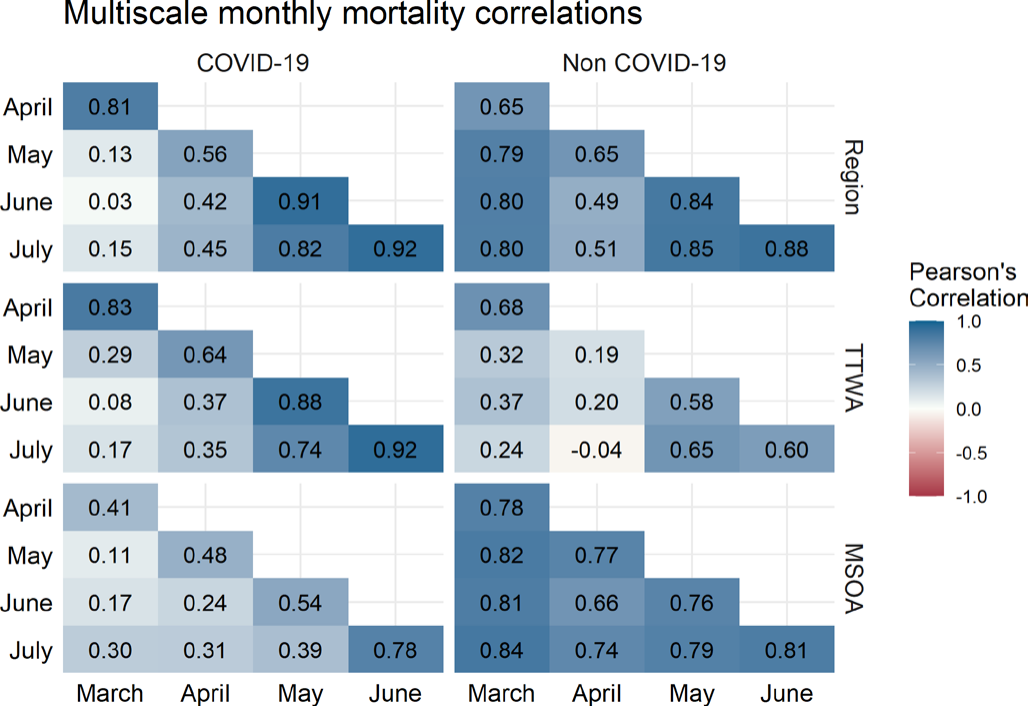
Correlation between month-specific structured residuals at three administrative scales for COVID-19 mortality (left) and non-COVID-19 mortality (right) after adjusting for local deprivation. Colour indicates magnitude of correlation. MSOA, Middle-Layer Super Output Area; TTWA, Travel to Work Area.

Having established spatial patterns of mortality inequality, we investigate to what degree these inequalities are explained by contextual deprivation. Number of care homes and decomposed IMD estimates were all interacted with month. Results from fully adjusted models are displayed in figures 4 and 5. Fixed effects estimates can be seen in online supplemental table 2.

Figure 4 demonstrates that adjusting for IMD does not substantively change the variance structure of the model. The COVID-19 mortality MRR in July for MSOA is 2.02 (95% CI 1.82 to 2.27). We still see area-level inequalities increase as national mortality rates decline. Regional MRR is still largest in July (MRR 2.41, 1.77–4.64). Non-COVID-19 mortality still displays far less geographical inequality. The correlation plots presented in figure 5 are similarly consistent with plots prior to the introduction of area deprivation, showing a strong regional clustering as nationwide mortality declines.

To test whether higher level regional mortality residuals are a function of London’s abnormality, fully adjusted models were rerun with London omitted (see online supplemental figures 7 and 8). Results suggest that some higher level regional inequality, particularly in March, may be driven by abnormally high age-standardised mortality observed in London, but that this does not appear to be the case in June/July. Omitting London reduces the magnitude of regional inequality to more in line with that of MSOA and TTWA. Omitting London also suggests that some of the temporal autocorrelation in regional non-COVID-19 mortality is driven by London, with smaller correlations in model SA1 than 2B.

To display how the importance of geographical context evolved over the period, figure 6 displays VPCs for each geographical level for COVID-19 and non-COVID-19 mortality over time from the unadjusted model (for deprivation-adjusted VPC estimates see online supplemental figure 9). In March and July, nearly half of the unexplained variation in MSOA-level COVID-19 mortality is due to higher level spatial context (July MSOA VPC=0.55). For non-COVID-19 mortality almost all variation is at local MSOA context across the study period (July MSOA VPC=0.98).

## Conclusions

Our results highlight that spatial inequality in COVID-19-related deaths at all scales is substantially higher than deaths due to other causes. Clearly, geography matters for COVID-19 mortality and it matters more than it does for mortality from other causes.

The extent to which geography matters has changed over time. After April, the chance of someone dying with COVID-19 varied by where they lived to an increasing extent until July. We propose these changes likely reflect structural factors affecting risk of exposure or infection for two reasons. First, phylogenetic evidence suggests aetiological evolution over the summer of 2020 impacted viral transmission, not disease progression.^12 13^ Thus, conditional on age structure, we anticipate mortality changes likely relate to changes in infection, not prognosis. Second, suppression measures make it unlikely that compositional migration patterns drove short-term mortality differences.

The geography of COVID-19 mortality is structured at different scales. While this is often implicitly acknowledged in debate, particularly around North-South divides, we believe this is the first study to attempt to quantify and apportion this. We find inequalities at each examined scale but find particularly large inequalities at the largest (GOR) scale. Our results are consistent with other findings on clustered introduction of the virus producing large initial spatial inequalities, driven partially by high mortality in London. Monthly VPCs illustrate the continued importance of spatial context for COVID-19 mortality over non-COVID-19 mortality, but that regional inequalities are more important when overall COVID-19 mortality is low. Excluding London, our findings remain consistent, with median comparisons between regions giving a twofold relative mortality increase by July (MRR=2.16, 1.59–4.41).

We adjust for IMD to explore how local, deprivation-related exposures affect the variance structure of the model, and find consistent with previous studies, deprived areas have greater COVID-19 mortality than non-deprived areas. We do not find deprivation meaningfully alters the variance structure, although IMD captures many deprivation exposures rather than our specific exposures of interest, which may dilute an anticipated effect.^31^ There is some evidence supporting initial misclassification of COVID-19 mortality, as MSOA non-COVID-19 MRR pre-empts that of the COVID-19 MRR before declining, and care homes predicting non-COVID-19 mortality in April, prior to the care home spike in COVID-19 mortality in May. There is strong evidence of emerging regional trends by July. Moreover, such inequalities are not explained by deprivation, care homes or age structure. Results suggest that a devolved geographical approach to COVID-19 support is likely useful. This approach must be multiscale with local community strategies embedded within regional frameworks and must consider pre-existing health and social inequalities.

Our study has several limitations. Our model considers geography in a strictly hierarchical sense, where we know in which TTWA/region each MSOA/TTWA is situated. It does not consider spatial contiguity/proximity and spatial networks. Clearly for infectious diseases, connections between and within areas are important and may explain some of the patterns we see here. Moreover, we cannot report on the importance of spatial context relative to within-MSOA, between-individual differences, as individual-level data are not available. If and when such data become available, a suite of more informative analyses will become possible.^32 33^

We propose that our study informs outcomes beyond COVID-19, as the modelling approach is of clear utility for studying other health outcomes. First, our approach can help advise where and how to apportion funding for tackling nationwide health inequalities. Assuming our goal is to reduce absolute inequalities, this approach informs how we might effectively do so by providing a structured framework for prioritising health inequalities between regions; between cities, towns or areas within regions; or between neighbourhoods within these cities, towns and areas.

Second, the modelling approach aims explicitly to distinguish policy-relevant systemic differences from chance variation. Models necessarily require assumptions about the degree of stochasticity which truly represents uncertainty, indeed phylogenetic evidence suggests UK SARS-CoV-2 evolution is a truly stochastic process.^13^ Spatial epidemiology, however, often fails to explicitly recognise chance variation as inherent in population health.^34^

Latent structural information contained in spatial data is being deployed across a wide range of geographical contexts to investigate spatial inequalities (eg, ref 35–39). Moreover, these data are providing important evidence in establishing effects of associated structural exposures.^16 40 41^ Our method is readily extendable to such contexts in the presence of: existing, administrative; or bespoke, researcher-imposed higher level spatial identifiers.

Continued monitoring of area-level mortality will be a fundamental piece of the evidence base helping mitigate the impacts of the COVID-19 pandemic. Mortality data at MSOA level are currently only available for the defined study period but analysing multiscale inequalities in mortality is critical to evaluating the impact and efficacy of viral suppression measures. Importantly, in periods of low overall mortality, we see large increases in regional inequalities in COVID-19 mortality. Regional inequalities in COVID-19 mortality may be of increasing importance as countries begin to emerge from lockdown and vaccine roll-out contributes to reduction in COVID-19 transmission and mortality. We suggest multiscale spatial inequalities which inform COVID-19 mortality, predominantly via exposure and infection, likely represent a key potential structural intervention for virus suppression.

**What is already known on this subject**

- Mortality resulting from COVID-19 has been strongly spatially patterned in the UK, with large regional inequalities observed and reported with the ‘second wave’ of infections in late 2020. Inequalities have been observed at both regional level and neighbourhood level, but no studies have investigated the relative importance of these levels of inequality simultaneously.

**What this study adds**

- Regional inequalities declined from an initial peak in April before increasing again in June/July. Within-region inequalities increased steadily from March until July. Strong regional mortality trends are evident by June/July as overall mortality declines, prior to wider reporting of regional differences in ‘second wave’. Analogous inequalities are not observed for non-COVID-19-related mortality, and are not explained by age structure, care homes or deprivation. As overall mortality declines, structural exposures at the regional level may present a key intervention target for reducing geographical inequalities in COVID-19 mortality.

## Data Availability

Data are available in a public, open-access repository. ONS COVID-19 mortality data are available here: https://www.ons.gov.uk/peoplepopulationandcommunity/birthsdeathsandmarriages/deaths/datasets/deathsinvolvingcovid19bylocalareaanddeprivation. ONS administrative geographical identifiers are available here: https://geoportal.statistics.gov.uk. ONS population health estimates are available here: https://www.ons.gov.uk/peoplepopulationandcommunity/populationandmigration/populationestimates/datasets/middlesuperoutputareamidyearpopulationestimates. ESRI care home estimates are available here: https://covid19.esriuk.com/datasets/e4ffa672880a4facaab717dea3cdc404_0. Equivalised UK IMD estimates are available here: https://data.bris.ac.uk/data/dataset/1ef3q32gybk001v77c1ifmty7x. 2019 England IMD estimates are available here: https://www.gov.uk/government/statistics/english-indices-of-deprivation-2019. All codes to reproduce analyses are available here: www.github.com/zimbabwelsh/covid_mort_spatseg.
