# Supplementary material for: Interrogating structural inequalities in COVID-19 mortality in England and Wales
Source: J Epidemiol Community Health. 2021 Jul 20;75(12):1165–71. doi: 10.1136/jech-2021-216666 (PMC8295019; doi:10.1136/jech-2021-216666)
Supplement: Supplementary data [file jech-2021-216666supp001.pdf]

**Supplementary Materials**

*Model Specification*

Equation 1, below, sets out our approach. For clarity this is illustrated with a simpler version of the model with only two months (March and April) and two geographic scales (MSOA and LAD). In practice we include 5 months and 4 geographical scales as the equation below is readily extendable . Although the data for this model have two levels, MSOA and LAD, the specified model has an extra pseudo-individual level, level 1, though in reality the units at this level are identical to the MSOAs at level 2. The reason for this extra level is it enables us to include two sources of variation in the model; stochastic variation in the counts following a Poisson distribution and the overdispersion identified above on top of this which we consider to be systematic inequality (Leckie et al. 2020). The variance at level 1 is constrained to be equal to the underlying rate for each group, as in a Poisson distribution, while the extra-Poisson variance at level 2 is then estimated in the model giving a measure of systematic inequality at the MSOA level.

$$O_{ijk} \sim Poisson(\pi_{ijk})$$

$$Log_e \pi_{ijk} = Log_e(E_{ijk}) + \beta_{1jk} March + \beta_{2jk} April$$

$$\beta_{1jk} = \beta_1 + v_{1k} + u_{1jk}$$

$$\beta_{2jk} = \beta_2 + v_{2k} + u_{2jk}$$

$$\begin{bmatrix} v_{1k} \\ v_{2k} \end{bmatrix} \sim N\left(0, \begin{bmatrix} \sigma^2{}_{v1} & \\ \sigma_{v12} & \sigma^2{}_{v2} \end{bmatrix}\right)$$

$$\begin{bmatrix} u_{1jk} \\ u_{2jk} \end{bmatrix} \sim N\left(0, \begin{bmatrix} \sigma^2{}_{u1} & \\ \sigma_{u12} & \sigma^2{}_{u2} \end{bmatrix}\right)$$

$$\mathrm{Var}(O_{ijk} | \pi_{ijk})$$

In the model O_ijk is a long stacked vector of observed counts for individual, i in MSOA, j, in Local Authority District, k with repeated observations for each month in the model. The observed counts are treated as coming from a Poisson distribution with mean rate, π_ijk. The natural log of this underlying rate is modelled.

March and April are dummy variables which indicate which month each count belongs to. E_ijk is the expected count for each MSOA in each month if deaths per unit of population were evenly distributed across MSOAs, or in other words if there was no inequality. The natural log of this is included in the model as an offset, with a coefficient constrained to 1. β1 and β2 give the log average (median) rate across MSOAs for March and April respectively.

Returning to the description of the model $u_{1jk}$ and $u_{2jk}$are, therefore, differences from the expected rate for MSOA$j$ for March and April respectively, while $v_{1k}$ and $v_{2k}$are the same the same for LADslevel. Positive values denote a higher than expected log rate for that geographical unit, while negative values denote a lower than expected log rate. These differences at each level are assumed to come from a joint normal distribution and can be summarized by variances $\sigma^2{}_{u1}$, $\sigma^2{}_{u2}$ , $\sigma^2{}_{v1}$, and $\sigma^2{}_{v2}$ ,respectively. It is these variances which measure the inequality and are the parameters of interest. For example $\sigma^2{}_{u1}$is a measure of inequality for March at the MSOA level.

Finally, $\sigma_{u12}$ and $\sigma_{v12}$ are the covariances, between the two groups at the two levels which can be standardised to give a correlation ranging between -1 and 1. These allow us to see if it is the same MSOAs and LADs which have high deaths in March are also the same MSOAs which have high deaths in April.

We also derive VPCs for the 3-level overdispersed, Poisson specification using the formulation in Leckie et al. (2020). VPCs are calculated for month specific intercept terms, so are for an area of average age structure, with zero care homes, and where relevant of average deprivation (both within and between areas).

*Supplementary Analysis*

| *ONS Reported Mortality* | COVID-19 Mortality | Non-COVID-19 Mortality |
|---|---|---|
| March | 4759 | 48 563 |
| April | 30 634 | 50 611 |
| May | 11 932 | 38 114 |
| June | 3391 | 34 772 |
| July | 1052 | 35 097 |

*Supplementary Table 1: Monthly Mortality Counts for COVID-19 and Non-COVID-19 causes of death.*

Fixed effect results allow us to investigate more clearly the following two questions of interest:

1. Is the effect of local-area deprivation consistent both within and between higher level spatial units?
2. Do observed mortality rate differences for care homes and MSOA age structures exist net of deprivation?

*Supplementary Table 2: Fixed Effect Coefficients for Models 2A and 2B, reflecting mortality risk ratios for COVID-19 and Non COVID-19 mortality.*

| | 2A: COVID-19 Mortality | | | 2B: Non-COVID-19 Mortality | | |
|---|---|---|---|---|---|---|
| | Rate Ratio | 2.5% CI | 97.5% CI | Rate Ratio | 2.5% CI | 97.5% CI |
| March | 0.442 | 0.264 | 0.756 | 0.875 | 0.823 | 0.934 |
| April | 0.528 | 0.381 | 0.691 | 0.801 | 0.736 | 0.875 |
| May | 0.550 | 0.382 | 0.732 | 0.902 | 0.819 | 0.996 |
| June | 0.493 | 0.267 | 0.802 | 0.914 | 0.838 | 1.002 |
| July | 0.444 | 0.205 | 0.831 | 0.931 | 0.852 | 1.022 |
| MSOA % 25-44 | 0.999 | 0.994 | 1.003 | 1.000 | 0.998 | 1.002 |
| MSOA % 45-64 | 1.022 | 1.015 | 1.028 | 1.023 | 1.020 | 1.026 |
| MSOA % 65-74 | 0.943 | 0.931 | 0.954 | 0.983 | 0.978 | 0.987 |
| MSOA % 75+ | 1.124 | 1.113 | 1.135 | 1.091 | 1.087 | 1.095 |
| Carehomes*Mar | 1.022 | 1.008 | 1.036 | 1.039 | 1.035 | 1.043 |
| Carehomes*Apr | 1.063 | 1.055 | 1.071 | 1.059 | 1.053 | 1.063 |
| Carehomes*May | 1.082 | 1.071 | 1.093 | 1.037 | 1.034 | 1.042 |
| Carehomes*Jun | 1.050 | 1.034 | 1.066 | 1.033 | 1.027 | 1.037 |
| Carehomes*Jul | 1.029 | 1.004 | 1.054 | 1.027 | 1.023 | 1.031 |
| RegIMD*Mar | 1.097 | 0.631 | 1.822 | 1.052 | 0.990 | 1.131 |
| RegIMD*Apr | 1.168 | 0.861 | 1.519 | 1.067 | 0.980 | 1.164 |
| RegIMD*May | 1.197 | 0.904 | 1.613 | 1.053 | 0.956 | 1.178 |
| RegIMD*Jun | 1.210 | 0.777 | 2.098 | 1.061 | 0.969 | 1.176 |
| RegIMD*Jul | 1.135 | 0.626 | 2.291 | 1.052 | 0.962 | 1.168 |
| T-RIMD*Mar | 1.148 | 1.107 | 1.190 | 1.116 | 1.105 | 1.129 |
| T-RIMD*Apr | 1.101 | 1.077 | 1.125 | 1.107 | 1.094 | 1.120 |
| T-RIMD*May | 1.020 | 0.990 | 1.051 | 1.107 | 1.095 | 1.120 |
| T-RIMD*Jun | 1.009 | 0.963 | 1.057 | 1.113 | 1.100 | 1.125 |
| T-RIMD*Jul | 1.046 | 0.974 | 1.122 | 1.104 | 1.091 | 1.117 |

| | | | | | | |
|---|---|---|---|---|---|---|
| M-TIMD*Mar | 1.097 | 1.062 | 1.135 | 1.008 | 1.007 | 1.010 |
| M-TIMD*Apr | 1.121 | 1.100 | 1.142 | 1.006 | 1.004 | 1.007 |
| M-TIMD*May | 1.101 | 1.073 | 1.131 | 1.007 | 1.006 | 1.009 |
| M-TIMD*Jun | 1.099 | 1.055 | 1.143 | 1.007 | 1.005 | 1.009 |
| M-TIMD*Jul | 1.116 | 1.047 | 1.190 | 1.007 | 1.005 | 1.008 |

Supplementary Table 2 presents the fixed effect estimates from models 2A and 2B. Month estimates are predicted mortality rate ratio for an MSOA with an average age structure, of average deprivation (both regionally and locally) with zero care homes. Estimates for age structure illustrate that mortality increases with local proportion of ageing population, except for the instance where MSOAs have high levels of 65-74 year olds, which predicts lower mortality. This is likely indicative of areas with higher proportions of retiree populations being in more affluent areas.

We are primarily interested in the estimates for IMD. Estimates are imprecise, but evidence consistently points to higher mortality from both COVID-19 and non-COVID-19 in more deprived locations, both between and within MSOAs. Despite being consistently positive, the strength of the relationship between deprivation and mortality is not consistent over different spatial scales. The effect of regional IMD on COVID-19 mortality is consistently higher than it is for non-COVID mortality, although, as we would expect seeing the rest of the results, this is more strongly the case in months where COVID-19 mortality is lower. In months where COVID-19 mortality was high overall, spatial clustering of deaths was lower. We see this peak in June. Similarly within-region, between TTWA IMD predicts excess mortality strongly in March – but this lessens over the period. Between MSOA, within TTWA deprivation predicts excess COVID-19 mortality fairly consistently over the period, and far more strongly than it predicts excess non-COVID-19 mortality.

Care homes consistently predict excess COVID-19 mortality, peaking in May. Care homes most strongly predict non-COVID death in April before declining. This, taken with the negative residual correlation between April Non-COVID deaths and other months may indicate misclassification in COVID-19 deaths in care homes during April.

*Supplementary Analysis 1*

*Model SA1*, Replicating Model 2A with LADs replacing TTWAs

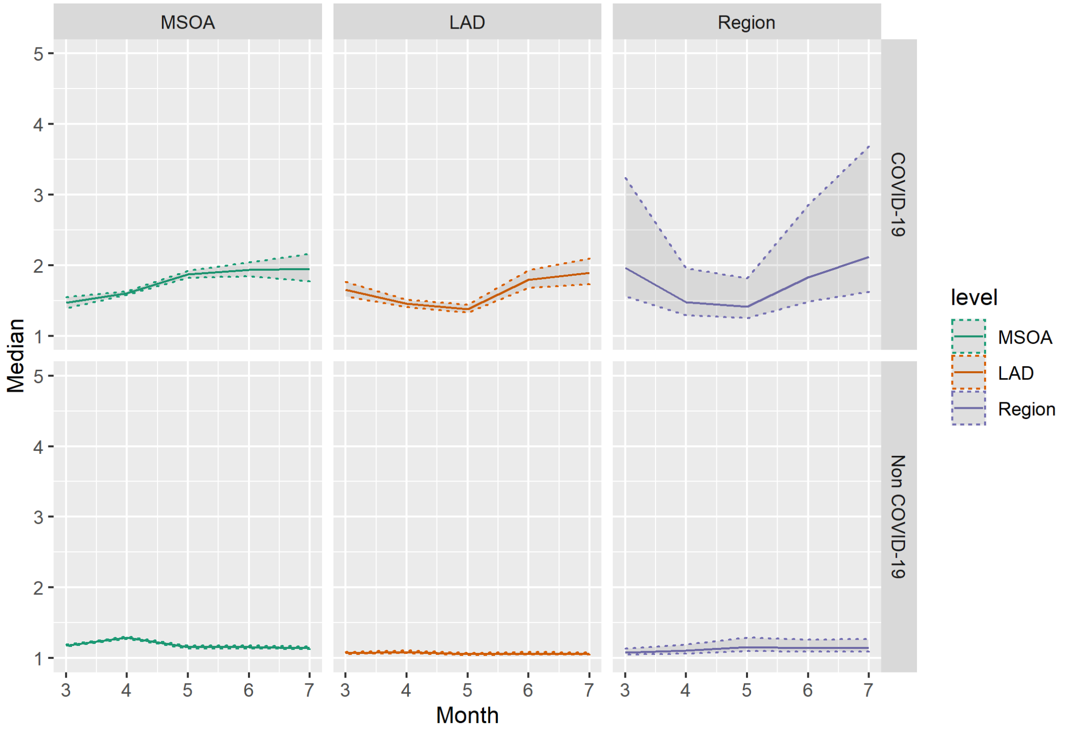


*Supplementary Figure 1A (top) and 1B (bottom): Estimates of Median COVID-19 (1A) and Non-COVID (1B) Median Mortality Rate ratio including LAD instead of TTWA across spatial scales after inclusion of local area deprivation. Shaded areas indicate 2.5th and 97.5th percentile credible intervals of posterior parameter.*

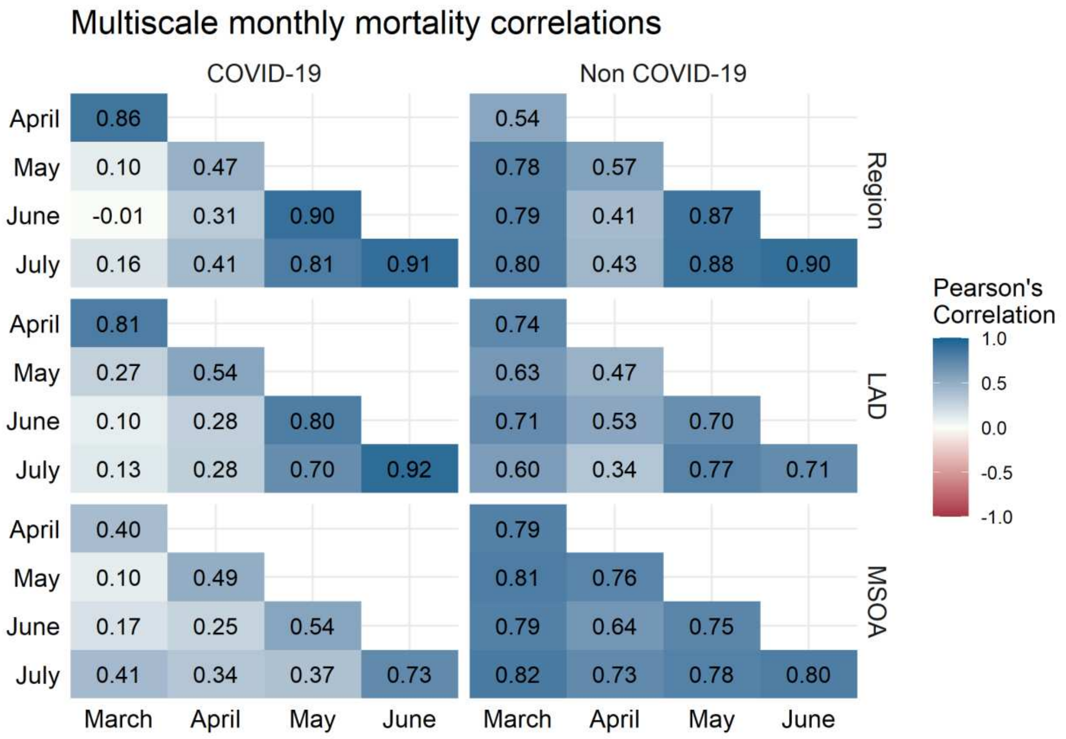


*Supplementary Figure 2: Correlations between month-specific structured residuals at 3 spatial scales for COVID-19 mortality (left) and non-COVID mortality (right) after adjusting for local deprivation, with LAD instead of TTWA.*

Supplementary Analysis 2 – including STP alongside TTWA

*Model SA3* – Replicating Model 2, with MSOA cross-classified into TTWA and STP:

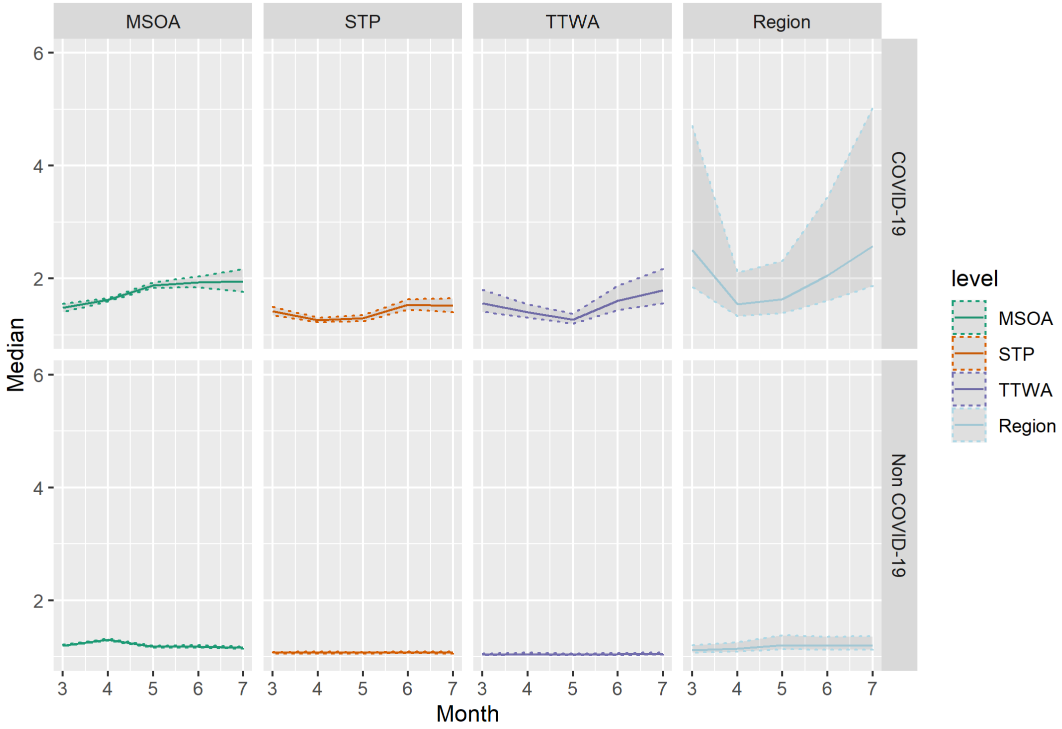


*Supplementary Figure 3A (top) and 3B (bottom): Estimates of Median COVID-19 (3A) and Non-COVID (3B) Median Mortality Rate Ratio including cross-classified STP level across spatial scales after inclusion of local area deprivation. Shaded areas indicate 2.5th and 97.5th percentile credible intervals of posterior parameter.*

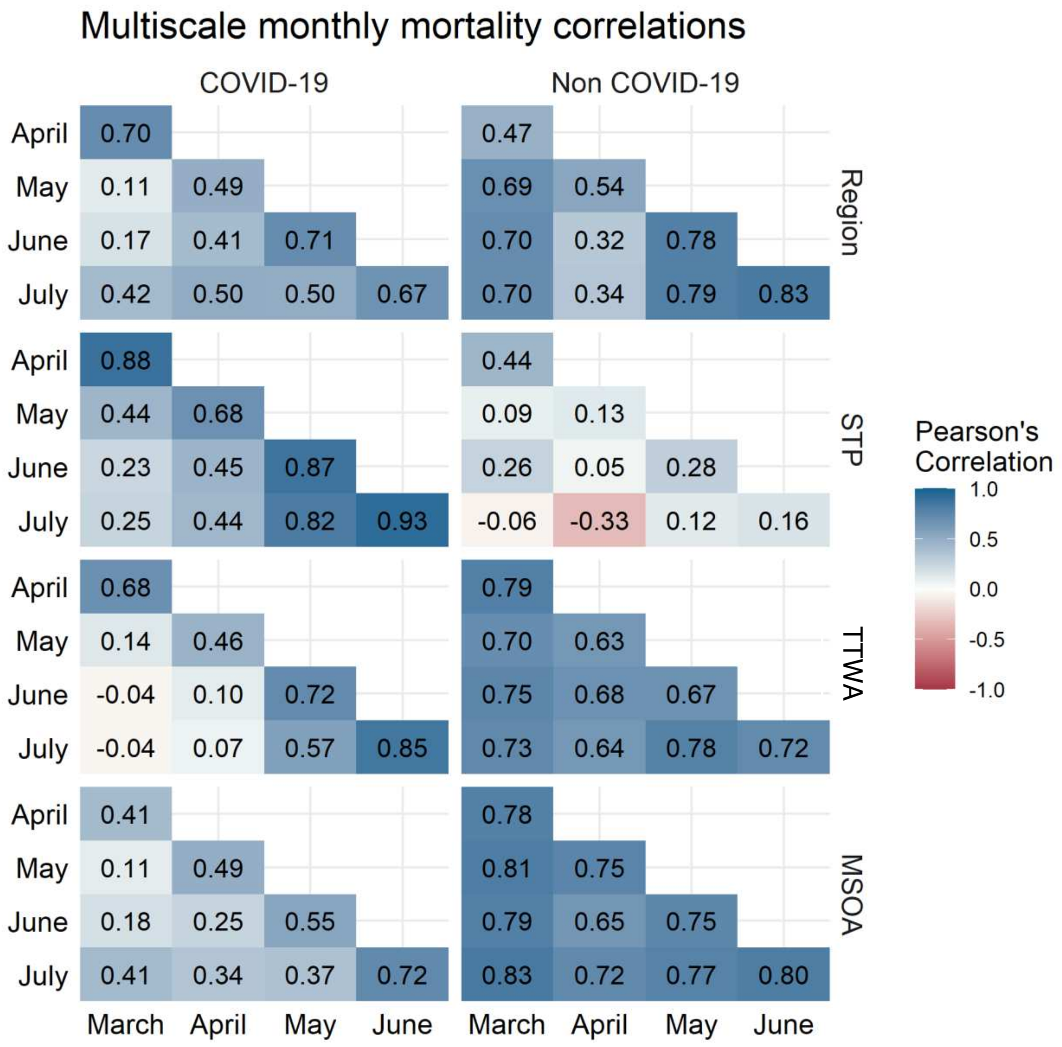


*Supplementary Figure 4: Correlations between month-specific structured residuals at 4 spatial scales for COVID-19 mortality (left) and non-COVID mortality (right) after adjusting for local deprivation, including the cross-classified level of STP.*

*Supplementary Analysis 3*

*Model SA3* – Replicating Model 2, with IMD updated to 2019 values using England specific ONS data. Welsh regions omitted from results:

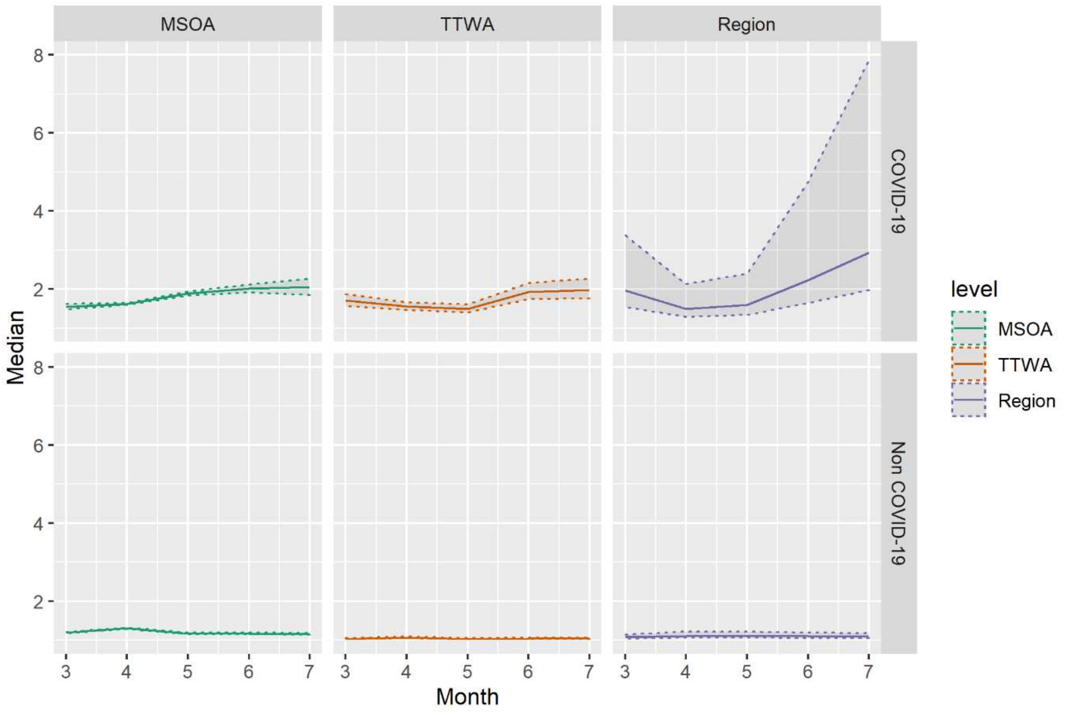


*Supplementary Figure 5A (top) and 5B (bottom): Estimates of Median COVID-19 (5A) and Non-COVID (5B) Median Mortality Rate Ratio including updated England-specific local area deprivation (2019). Shaded areas indicate 2.5th and 97.5th percentile credible intervals of posterior parameter.*

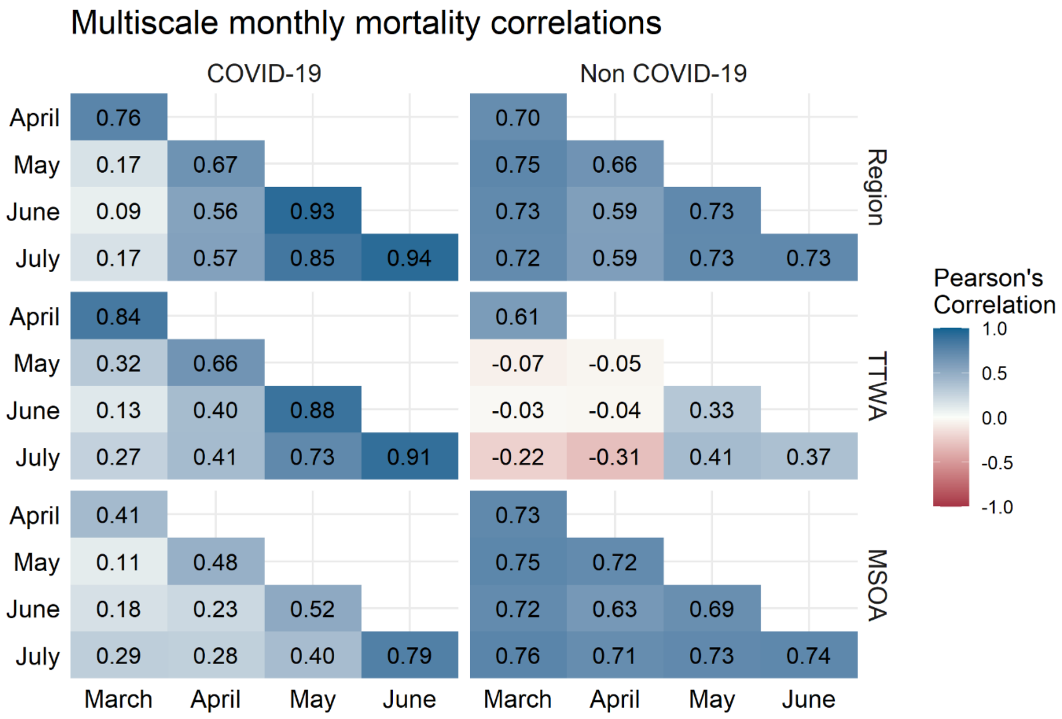


*Supplementary Figure 6: Correlations between month-specific structured residuals at 4 spatial scales for COVID-19 mortality (left) and non-COVID mortality (right) after adjusting for local deprivation, including the cross-classified level of STP.*

*Supplementary Analysis 4*

*Model SA4* – Replicating Model 2, with London omitted from results:

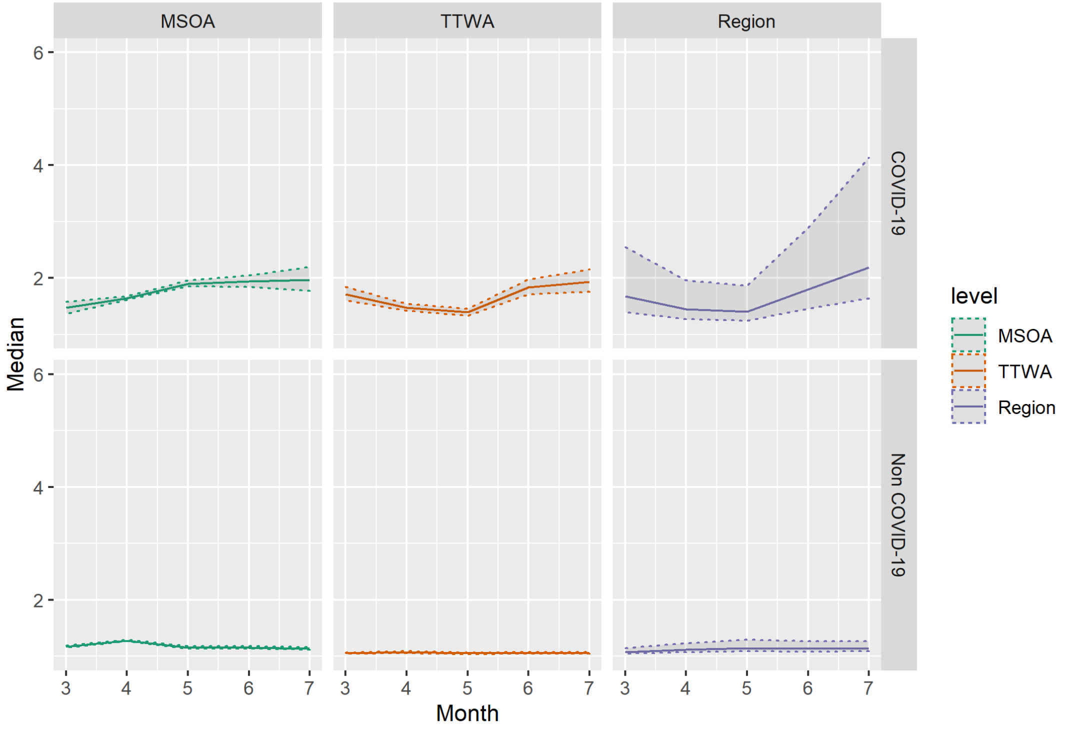


*Supplementary Figure 7A (top) and 7B (bottom): Estimates of Median COVID-19 (7A) and Non-COVID (7B) Median Mortality Rate Ratio omitting region of London across spatial scales after inclusion of local area deprivation. Shaded areas indicate 2.5th and 97.5th percentile credible intervals of posterior parameter.*

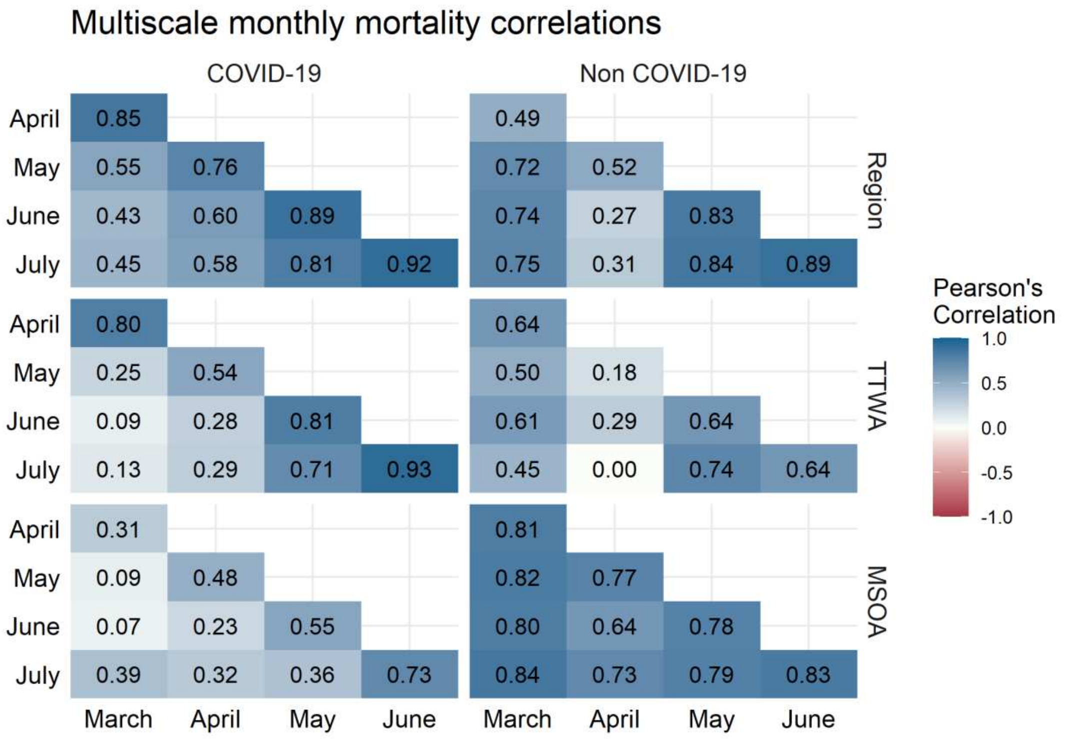


*Supplementary Figure 8: Correlations between month-specific structured residuals at 3 spatial scales for COVID-19 mortality (left) and non-COVID mortality (right) after adjusting for local deprivation, omitting region of London.*

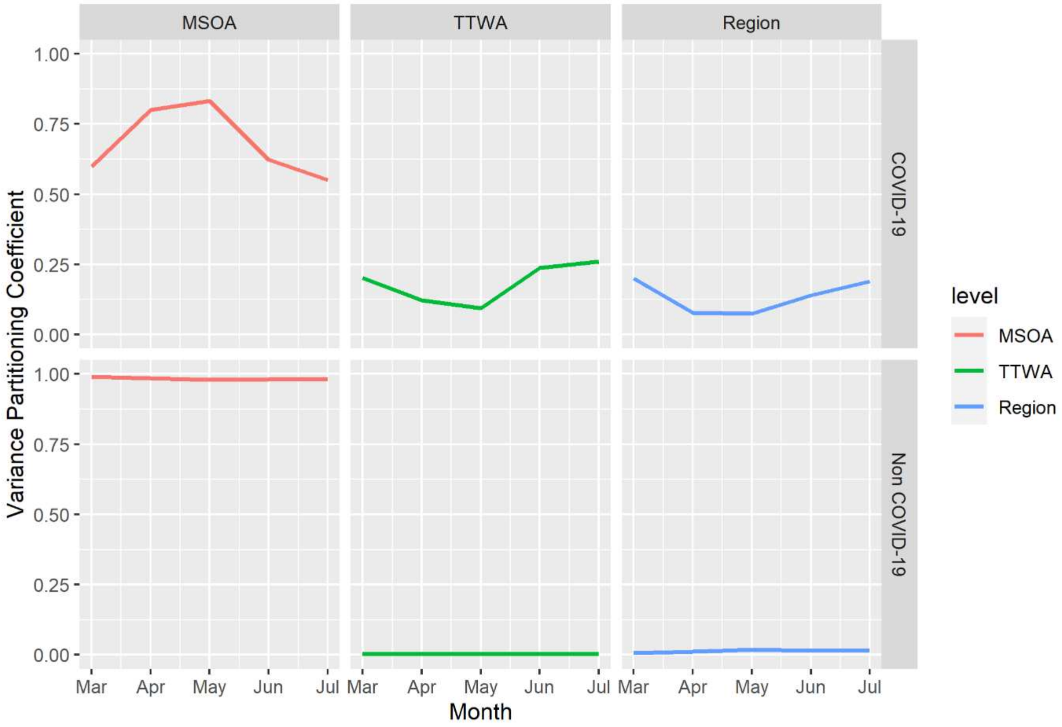


*Supplementary Figure 9: Local Deprivation-adjusted Model Monthly Variance Partitioning Coefficients (VPCs) for 3 administrative scales for COVID-19 related mortality (top) and Non-COVID mortality (bottom). VPC units are proportions*
